# IL-10 regulates adult neurogenesis by modulating ERK and STAT3 activity

**DOI:** 10.3389/fncel.2015.00057

**Published:** 2015-02-25

**Authors:** Leticia Pereira, Miriam Font-Nieves, Chris Van den Haute, Veerle Baekelandt, Anna M. Planas, Esther Pozas

**Affiliations:** ^1^Unit of Brain Ischemia, Institut d’Investigacions Biomèdiques August Pi i Sunyer, BarcelonaSpain; ^2^Department of Brain Ischemia and Neurodegeneration, Institute of Biomedical Research of Barcelona, Consejo Superior de Investigaciones Científicas, BarcelonaSpain; ^3^Laboratory for Neurobiology and Gene Therapy, Faculty of Medicine, KU Leuven, LeuvenBelgium; ^4^Leuven Viral Vector Core, KU Leuven, LeuvenBelgium

**Keywords:** adult neurogenesis, SVZ, IL-10, cell signaling, ERK, STAT3

## Abstract

The adult subventricular zone (SVZ) contains Nestin+ progenitors that differentiate mainly into neuroblasts. Our previous data showed that interleukin-10 (IL-10) regulates SVZ adult neurogenesis by up-regulating the expression of pro-neural genes and modulating cell cycle exit. Here we addressed the specific mechanism through which IL-10 carries out its signaling on SVZ progenitors. We found that, *in vitro* and *in vivo,* IL-10 targets Nestin+ progenitors and activates the phosphorylation of ERK and STAT3. The action of IL-10 on Nestin+ progenitors is reversed by treatment with a MEK/ERK inhibitor, thus restoring neurogenesis to normal levels. Silencing STAT3 expression by lentiviral vectors also impaired neurogenesis by blocking the effects of IL-10. Our findings unveil ERK and STAT3 as effectors of IL-10 in adult SVZ neurogenesis.

## INTRODUCTION

Postnatal neurogenesis takes place in restricted regions or niches in the adult brain. The SVZ lining the LVs is one of the main neurogenenic niches in the adult brain. The niche is composed by supporting cells, the vasculature and three progenitor cell types: slow-cycling glial-like NSCs or type B cells (GFAP+); TACs or type C cells (Ki67+, and Mash1+), and the more differentiated neuroblasts (type A cells; PSA-NCAM+; DCX+; TUBB3+) that migrate over long distances through the RMS to reach the olfactory bulb, where they finally become mainly GABAergic interneurons (Lois and Alvarez-Buylla, 1994; Doetsch and Alvarez-Buylla, 1996; Doetsch et al., 1997; Merkle et al., 2004; Ihrie et al., 2011). Nestin labels type B and C cells and a sub-population of immature committed neuroblasts (Doetsch et al., 1997; Perez-Asensio et al., 2013). The SVZ niche is unique in spatial localization and molecular characteristics. The relationships between the different cell types, the cerebrospinal fluid (CSF), and the vasculature modulate the molecular signals that regulate self-renewal, proliferation, the identity of VZ-SVZ-derived progeny, the integration of some intrinsic mechanisms (Guillemot, 2007; Lim et al., 2009; Ihrie et al., 2011).

Interleukin-10 (IL-10) is a general anti-inflammatory molecule that contributes to maintaining the pro- and anti-inflammatory balance in the body (Pestka et al., 2004; Saraiva and O’Garra, 2010; Ouyang et al., 2011). Recently, we demonstrated a new physiological role of this cytokine as a relevant factor that regulates postnatal neurogenesis. We deciphered how IL-10 targets the population of Nestin+ progenitors located in the dorsal SVZ, where it regulates the expression of undifferentiated neural progenitor markers, cell cycle activity, and the production of new neuroblasts (Perez-Asensio et al., 2013).

Here we aimed to identify the specific intracellular mechanism through which IL-10 acts specifically on adult Nestin+ progenitors. Our results show that IL-10 regulates the activation of ERK and STAT3 in Nestin+ progenitors and that this activity is required for IL-10 to exert its actions on neural progenitors.

## MATERIALS AND METHODS

### RECOMBINANT PROTEINS, REAGENTS AND ANIMALS

Interleukin-10 was purchased from Prepotech (Rocky Hill, CT, USA) and R&D (Minneapolis, MN, USA), and U0126 was from Merck-Millipore (Darmstadt, Germany). Mice (C57BL/6) and rats (Wistar) were obtained from Charles River (Lyon, France). All animals were male and age-matched. Animals work was carried out in accordance with the European Community Council Directives on animal welfare and according to the local regulations. Every effort was made to minimize animal suffering.

### PRIMARY CULTURES

Cell cultures were performed from postnatal brains of rats (P7–P9) as previously described (Perez-Asensio et al., 2013). Briefly, the SVZ was gently microdissected mildly trypsinized and platted in poly-L-lysine coated plates and cultured in DMEM/F12 supplemented with B27 (Life Technologies, Paisley, UK) in the presence or absence of IL-10 (50 ng/ml). Lentiviral vector production and concentration was performed as described (Geraerts et al., 2006). Transduction of primary cells was performed during 4–6 h with 1 × 10^6^ transducing units (TU)/ml. Cultures were fixed in 4% paraformaldehyde (PFA), permeabilized with 0.1% triton and stained as below. Nuclei were routinely counterstained with Hoechst.

### BIOCHEMISTRY

Western blotting of primary cultures and tissue samples were processed as previously described (Perez-Asensio et al., 2013). Membranes were then incubated with the following antibodies: phosphorylated STAT1 and STAT3 (all from Cell Signaling Technology, 1:1000); DCX (Cell Signaling Technology 1:1000; Santa Cruz Biotechnology, Santa Cruz, CA, USA 1:500), Nestin (1:500, either from Merck-Millipore or BD Bioscience San Jose, CA, USA), Musashi (Millipore, 1:500), Mash1 (BD Bioscience, 1:500), total STAT-1 (BD transduction, 1:1000), total STAT-3 (BD Bioscience, 1:1000), Notch-ICD (Abcam, Cambridge, UK; 1:500), Numb (Abcam, 1:500); and Tubulin (Sigma, 1:50000), or Actin (Sigma, 1:50000) as loading controls.

### IMMUNOFLUORESCENCE

For immunofluorescence see procedure described Perez-Asensio et al. (2013). The list and dilution of primary antibodies was: pSTAT3_ser727_ (1:500), pERK1/2 (1:500), BIII-Tubulin (1:1000), DCX (1:1000), Nestin (1:200), Ki67 (Leica Microsystems, Wetzlar, Germany 1:1000), BrdU (AbCam, 1:400), Olig2 (Millipore, 1:400), GFAP (DAKO, 1:2000). Staining on cell cultures was visualized with a Leica CTR400-DMI400B inverted microscope or Leica DM5500Q confocal microscope. Photographs were taken by a DFC300FX camera from Leica. Quantification was carried out using Leica Application Suite (LAS-Leica) or ImageJ (NIH) software. The number of positive cells for each experimental assay was expressed as percentage of the total number, and/or referred as percentage of the control.

### INTRACEREBROVENTRICULAR SUBSTANCE INFUSION IN THE MOUSE BRAIN

The administration of the IL-10 (50 ng/ml) and/or U0126 (25 μM) at a low flow rate of 0.5 μl/h during 7 days in the third ventricle was carried out by continuous infusion with an Alzet^®^ osmotic minipump (model 1007D) and Alzet^®^ Brain Infusion Kit 3 (DURECT Corporation, Cupertino, CA, USA); see procedure in Perez-Asensio et al. (2013). The delivery of saline as the vehicle or IL-10 to the third ventricle was achieved by inserting the cannula 1.7 mm depth from the brain surface at -0.1 mm posterior, and 0.6 lateral coordinates (Franklin and Paxinos, 1997), after exposing Bregma. The contralateral (ctr) hemisphere (left) was always considered for histological analysis and some ipsilateral hemispheres were used for biochemical studies.

### INTRACEREBRAL ADMINISTRATION OF LENTIVIRAL VECTORS

Some pump-implanted mice received, during the same surgery operation, lentiviral particles (1 ul with 1 × 10^6^ TU) in the ventral striatum adjacent to the LV of left hemisphere at coordinates 1.7 mm depth from the brain surface at -0.1 mm posterior, and 0.6 lateral coordinates (Paxinos and Watson, 1998). Animals were sacrificed 7 days after operation.

### HISTOLOGY

See methodology in Perez-Asensio et al. (2013), briefly: mice (8 weeks) were perfused with 4% PFA and cryoprotected in 30% sucrose. Coronal sections (16 μm) were obtained and were collected in eight consecutive slice series. In all animals, the left (ctr) hemisphere was analyzed on histological sections.

Routinely, the total number of cells in dorsal SVZ was counted per section after TO-PRO3 staining (Invitrogen). Cell death was evaluated by the pattern of cleaved Spectrin by elecrophoresis and Western blotting and cleaved Caspase 3 immunostaining on sections (Cell Signaling, 1:150). After immunofluorescence analysis (see protocol above), brain sections were scanned and evaluated under a Leica DM5500Q confocal microscope. At least four consecutive sections from the same slice were evaluated for each staining and the number of positive cells for each section was counted after a Z projection by ImageJ or LAS (Leica application suite) software. For the SVZ analysis, pictures were taken between +1.10 and +0.38 mm from Bregma.

### STATISTICAL ANALYSIS

Analyses of significant differences between group means were performed using the two-tailed Student’s *t*-tests. In each case, *n* indicates the number of independent cultures or mice used. Statistical significance was considered always if *p* < 0.05.

## RESULTS

### IL-10 STIMULATES STAT3 PHOSPHORYLATION ON SVZ PROGENITORS

Our previous studies demonstrated that IL-10 receptor is present in Nestin+ progenitors of the adult SVZ and that IL-10 reduces neuronal differentiation by keeping progenitors in an active cycle and up-regulating pro-neural gene markers (Nestin, Sox1, Sox2, Musashi, Mash1; see Perez-Asensio et al., 2013). In the present study, we aimed to decipher the intracellular mechanism activated by IL-10 in SVZ progenitors.

**FIGURE 1:**
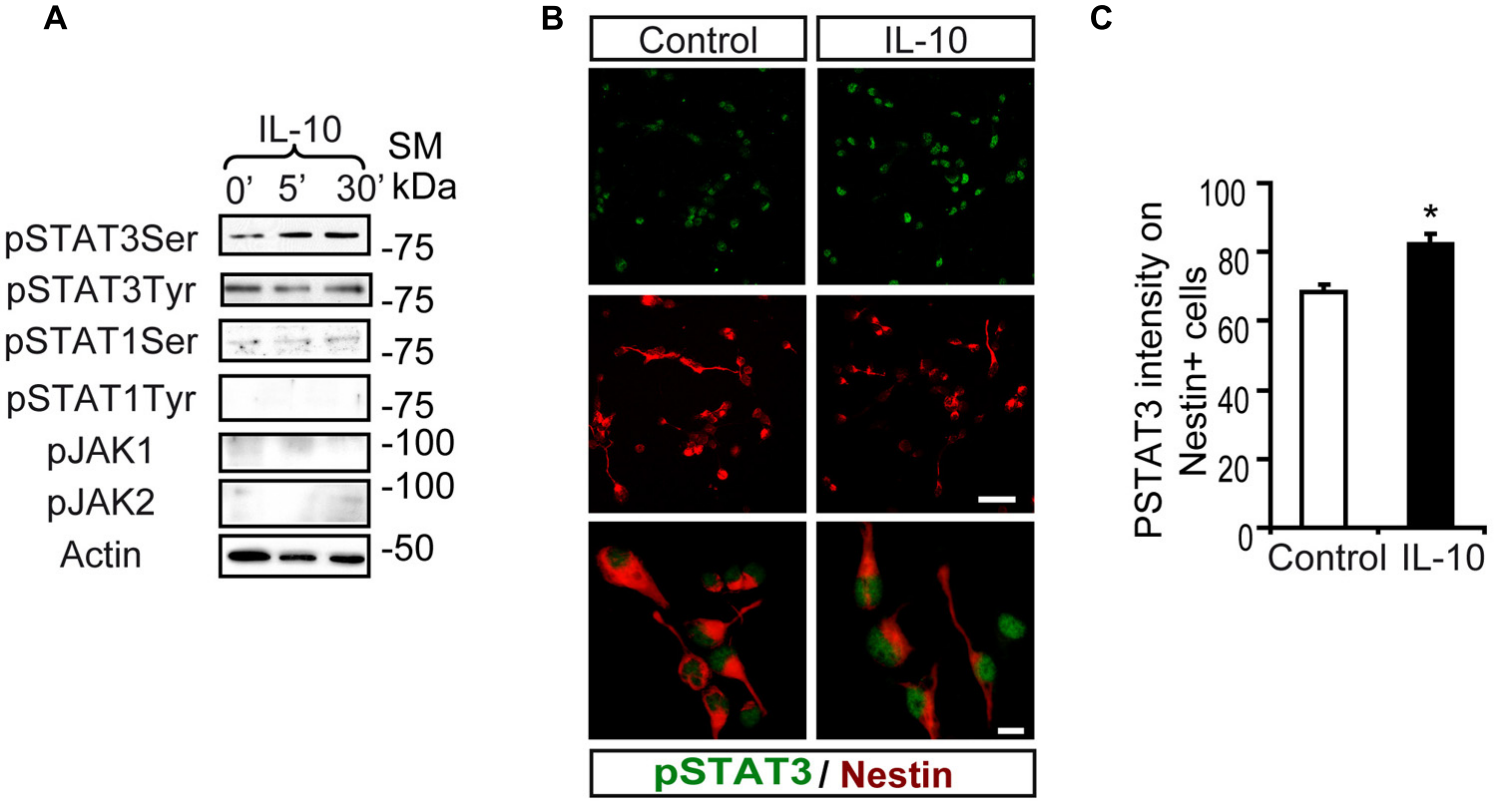
**Interleukin-10 increases serine STAT3 phosphorylation in Nestin+ SVZ progenitors. (A)** Phosphorylation of STAT3_Ser727_ was induced from 5 min after IL-10 (50 ng/mL) addition in primary cultures of the SVZ. Stimulation of pSTAT3_Tyr701_, STAT1, and JAK1 and JAK2 were not observed (*n* = 6 per time point). **(B)** Double-immunofluorescences stainings show that STAT3_Ser727_ immunoreactivity (green) was increased in Nestin+ progenitors (red) in IL-10 stimulated cultures (30 min after administration), compared with vehicle treatment (control). TO-PRO (blue) labeled all nuclei. (*n* = 5). **(C)** Histogram represents the nuclear immunoreactivity intensity of STAT3_Ser727_ per Nestin+ cells in control and after IL-10 stimulation. After IL-10 stimulation phosphorylation of STAT3_Ser727_ was exacerbated in Nestin+ progenitors (*n* = 4). Scale bar **(B)**, 30 μm. Data are represented as mean ± SEM. *P ≤ 0.05.

Interaction of IL-10 with IL-10R1 followed by JAK1-mediated tyrosine phosphorylation of STAT3 is required for IL-10 to exert its anti-inflammatory activity (Ouyang et al., 2011). Stimulation assays with IL-10 on SVZ-dissociated cultures showed a rapid phosphorylation of STAT3 on Ser727 (pSTAT3_ser727_), while pSTAT3_tyr705_ was not induced (**Figure 1A**). IL-10 did not stimulate the phosphorylation of STAT1 (either on Tyr701 or Ser727) or JAK family members (JAK1 and JAK2; **Figure 1A**). Double immunofluorescence analysis demonstrated that low levels of pSTAT3_ser727_ were constitutively present in the nuclei of SVZ neural cells and they did not change in the presence of the vehicle (experimental control; **Figures 1B,C**). In contrast, IL-10 increased the signal intensity of nuclear pSTAT3_ser727_ in Nestin+ progenitors (**Figures 1B,C**).

### ERK SIGNALING IS ACTIVATED BY IL-10 IN SVZ PROGENITORS *IN VITRO* AND *IN VIVO*

**FIGURE 2:**
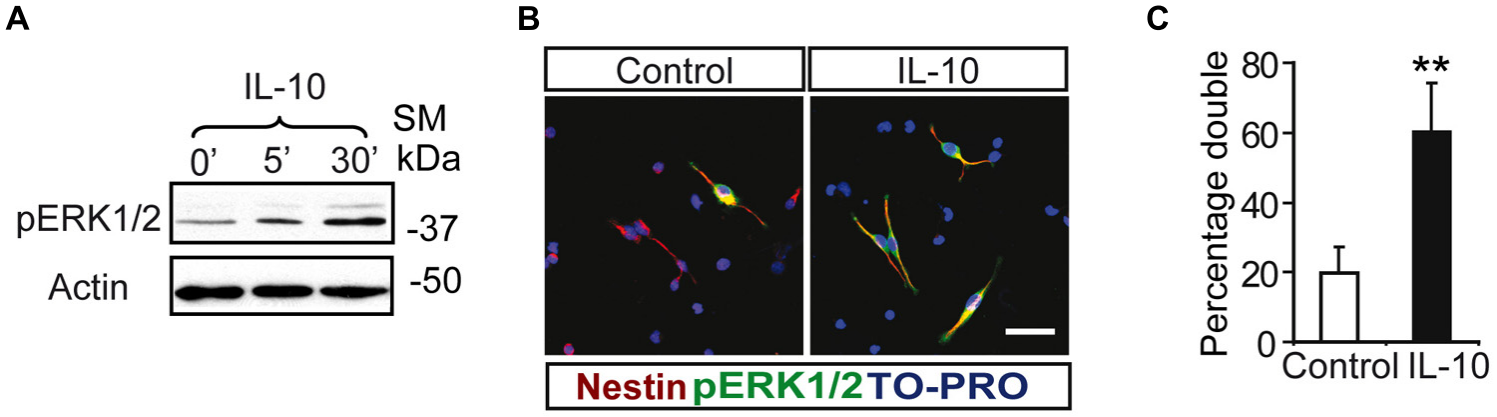
**Interleukin-10 induces the activation of ERK p42–44 specifically in Nestin+ progenitors on SVZ primary cultures. (A)** ERK1/2 Phosphorylation was induced from 5 min after IL-10 addition in primary cultures of the SVZ (*n* = 5). **(B)** Duble-immunofluorescences stainings show that the number of positive cells for phosphorylated ERK1/2 (green) is increased after IL-10 stimulation compared with control. ERK1/2 Phosphorylation is restricted to Nestin+ progenitors (red) in both control and under IL-10 presence. TO-PRO (blue) labeled all nuclei. **(C)** Histogram represents the percentage of double-labeled cells (pERK+/Nestin+) in cells that received the vehicle (control) or IL-10 for 30 min. After IL-10 stimulation the phosphorylation of STAT3_Ser727_ was activated in the majority of Nestin+ (*n* = 4). Scale bar, 30 μm. Data are represented as mean ± SEM. **P ≤ 0.01.

**FIGURE 3:**
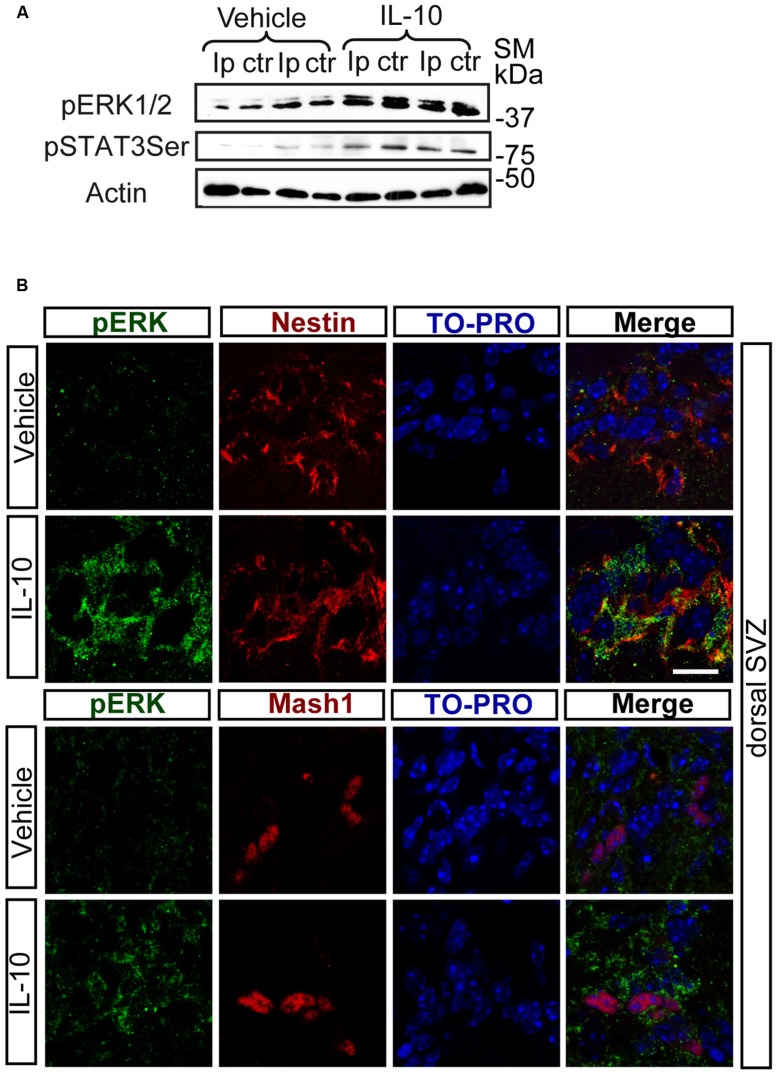
***In vivo* IL-10 induces the phosphorylation of ERK and STAT3. (A)** Phosphorylation of ERK1/2 and STAT3_ser727_ in the ipsilateral (Ip) and contralateral (ctr) SVZ niche of adult mice 30 min after they received an ICV injection of IL-10 (1 ul of 50 ηgr/ml; *n* = 3). **(B)** Pictures of dorsal SVZ after ICV IL-10 injection. ERK1/2 phosphorylation (green) takes place rapidly (30 min) in Nestin+ (red) and Mash1+ (red) progenitors cells in dorsal SVZ after *in vivo* IL-10 stimulation. To-pro (blue) stained all nuclei (*n* = 3). Scale bar, 30 μm.

Notch regulates cell survival in NSC cells by means of JAK-independent activation of STAT3 through AKT and mTOR phosphorylation (Androutsellis-Theotokis et al., 2006). In cell carcinomas, IL receptors activate STAT3 phosphorylation by JAK and/or MAPKs (Lee et al., 2006). Stimulation assays with IL-10 in SVZ cell cultures showed a rapid and strong induction of ERK1/2 phosphorylation (**Figure 2A**). pERK1/2 was present only in a small number of Nestin+ cells in the experimental control, as shown by double-immunofluorescence analysis. The acute presence of IL-10 induced a robust increase in the number of Nestin+ cells showing ERK1/2 phosphorylation (**Figures 2B,C**). The stimulation of ERK1/2 phosphorylation was restricted to Nestin+ progenitors and pERK1/2 was not detected in Nestin negative cells. We conclude that IL-10 targets specifically Nestin+ progenitors, in which promotes ERK1/2 activation. In contrast, AKT phosphorylation was not affected by the treatment with the cytokine (data not shown). The same situation was observed *in vivo* after acute treatment of adult mice with IL-10. A single ICV administration of IL-10 into the LV of the mice induced ERK1/2 and pSTAT3_ser727_ phosphorylation in the SVZ niche of both hemispheres, ctr and ipsilateral to the injection site (**Figure 3A**). Further studies on SVZ histological sections by double immunofluorescences revealed that IL-10 induced rapid pERK1/2 activation in Nestin+ progenitors and Mash1+ (TAPs) cells (**Figure 3B**). We were unable to assess the presence of pSTAT3_ser727_ *in vivo* since the antibodies available did not work on brain sections.

Systemic administration of IL-10 to the mice also activated intracellular pathways in the SVZ. Intravenous femoral administration of IL-10 induced rapid phosphorylation (30 min after administration) of ERK in the SVZ niche in a dose-dependent manner (Supplementary Figure S1)

All together, these data demonstrate that IL-10 activates ERK1/2 *in vivo* and *in vitro* and point to a subsequent activation of STAT3 on ser727 phosphorylation.

### ERK1/2 ACTS UPSTREAM OF STAT3 ON SVZ PROGENITORS AND MEDIATES THE BIOLOGICAL ACTION OF IL-10 BOTH *IN VIVO* AND *IN VITRO*

**FIGURE 4:**
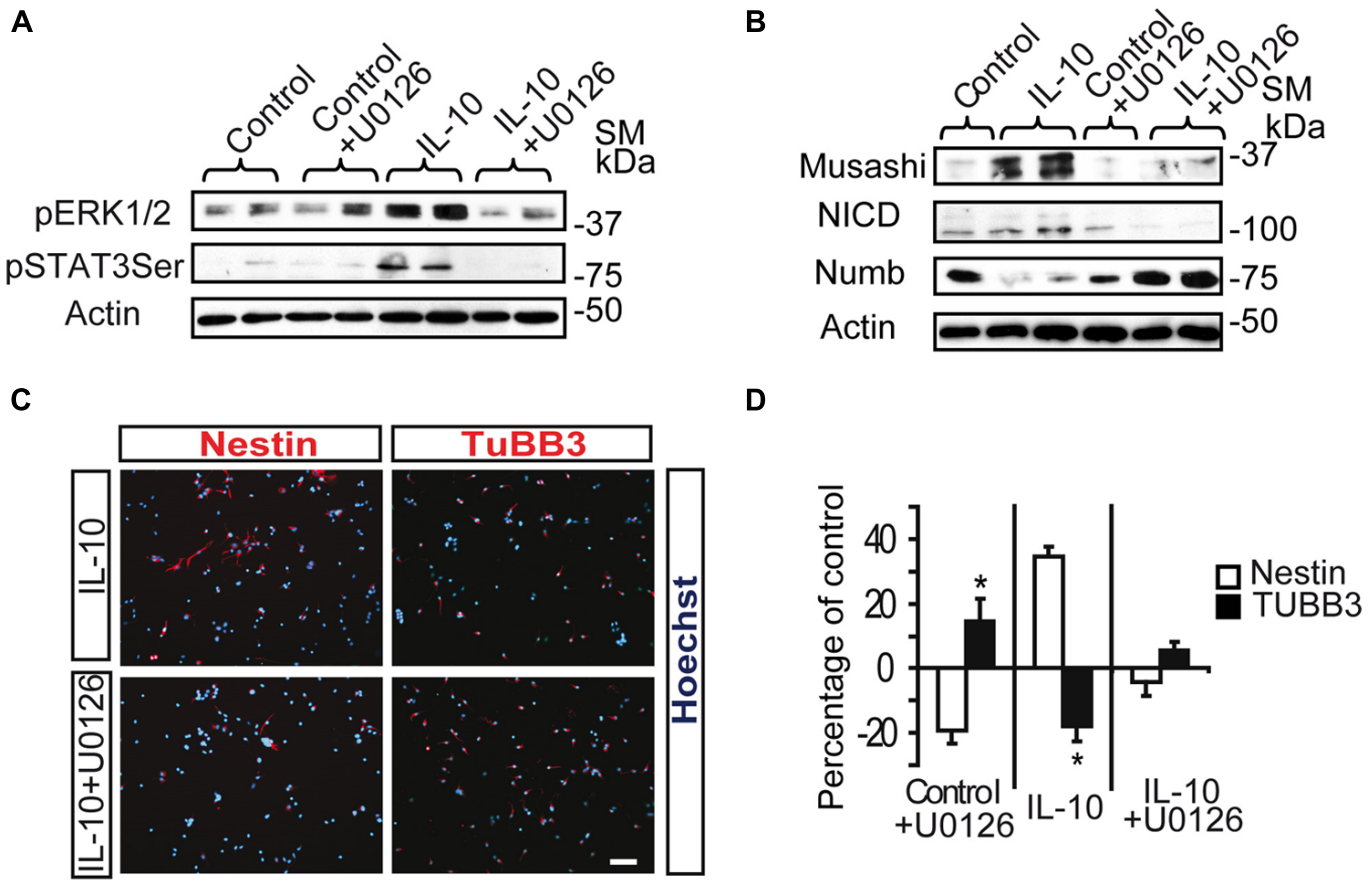
**ERK1/2 acts upstream of STAT3 and mediates IL-10 actions on SVZ progenitors. (A)** MAPK pathway inhibition by U0126 (25 μM) prevents STAT3_Ser727_ activation induced by IL-10. Actin was the loading control (*n* = 4). **(B)** Western blotting of neural protein expression after U0126 treatment during 48 h. UO126 prevents the up-regulation of Musashi, NICD and Numb induced by IL-10 (*n* = 4) **(C)** Pictures of dissociated cultures stain against Nestin and TUBB3 in the presence of IL-10 or IL-10+U0126. MEK inhibitor induced a decrease in the number of progenitors (Nestin+) and increases the number of neuroblasts (TUBB3+). Hoechst (blue) stained all nuclei. **(D)** Graph summarizes the effect of U0126 on the numbers of Nestin+ and TUBB3+ cells in the control situation and after IL-10 treatment. In control and IL-10-treated cultures U0126 reduced the presence of Nestin+ cells while it increased neuroblast number (TUBB3+). Values are expressed as the percentage of control (*n* = 5). Scale bar: 30 μm. Data are represented as the mean ± SEM. *P < 0.05.

Inhibition of the MAPK pathway by the MEK1/2 inhibitor U0126 prevented the induction of pSTAT3_ser727_ by IL-10 in SVZ primary cells (**Figure 4A**), thereby strongly suggesting that ERK1/2 activation is required for STAT3 phosphorylation. In a previous study we described that IL-10 modulates the undifferentiated state of SVZ neural progenitors by up-regulating neural markers, such as Nestin, Musashi, and NICD, while it decreases the expression of NUMB, a protein involved in neuronal differentiation (Perez-Asensio et al., 2013). Inhibition of the MAPK pathway abolished the IL10-mediated induction of the pro-neural markers Musashi and NICD and reduced NUMB expression (**Figure 4B**). Further analysis at the cellular level demonstrated that inhibition of ERK1/2 phosphorylation had considerable effects on the expression of neural markers. Indeed, U0126 induced a significant decrease in the number of Nestin+ cells, with a parallel increase in TUBB3+ cells (**Figures 4C,D**). This alteration was observed in control and IL-10-treated cultures, thus indicating that the ERK1/2 pathway not only is functional under basal conditions but it also mediates the biological effects induced by IL-10. Given that the ERK signaling pathway is involved in CNS specification of glial progenitors (Wang et al., 2012), we evaluated the effects of ERK inhibition on glial phenotype markers. In this regard, we observed that the number of GFAP+ cells was unaltered by the presence of the inhibitor in any condition, while the number of Olig2+ cells was reduced, independently of the presence or absence of IL-10 (Supplementary Figure S2).

**FIGURE 5:**
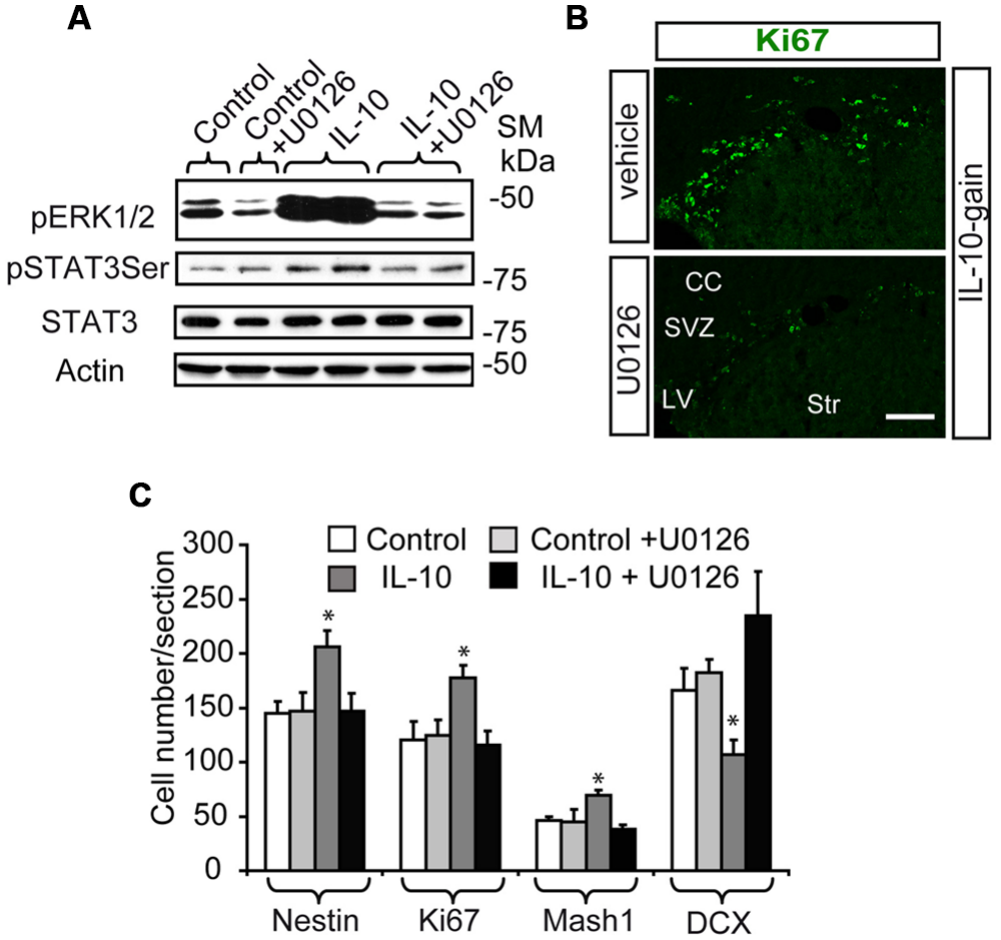
**The *in vivo* inhibition of ERK signaling abolish the up-regulation of TAPs induced by IL-10. (A)** Western blot shows that UO126 prevents the action of IL-10 on ERK1/2 and STAT3_ser727_ phosphorylation after 30 min of ICV administration. Expression of STAT3 and actin were unchanged (*n* = 3). **(B)** Pictures of SVZ niche in the presence of IL-10 plus inhibitor or respective vehicle. The presence of the inhibitor decreased the number of Ki67+ cells (green). **(C)** Histograms summarize the number of cells present in dorsal SVZ per section. As previously described Nestin+, Ki67+, and Mash1+ cells were increased and DCX + cells were reduced in dorsal SVZ on IL-10-treated animals [6]; and U0126 abolished the actions of the cytokine (*n* = 4). Scale bar: 50 μm. Data are represented as mean ± SEM. *P < 0.05.

In order to explore the relevance of the activation of ERK signaling by IL-10 *in vivo,* we infused mice via ICV administration with the MAPK inhibitor U0126 in combination with IL-10 (IL-10 gain) or vehicle. We used a very low dose of this drug to avoid secondary and unspecific effects of MAPK inhibition unrelated to IL-10 activity, since abnormalities in brain and cell fate specification of CNS progenitors have been reported in several contexts as a result of ERK signaling pathway alterations (Wang et al., 2012). As expected, the inhibitor attenuated the phosphorylation of both ERK1/2 and STAT3 on Ser727 after 30 min of IL-10 or vehicle treatment (**Figure 5A**). In contrast, the expression of total STAT3 and Actin (as a loading control) was unchanged. The administration of the inhibitor over several days did not affect cell survival or proliferation (see Materials and Methods). Cell number counts in brain sections were performed in order to examine the effects of the inhibitor on the population of undifferentiated progenitor cells. The MAPK inhibitor prevented the improvement in the number of cells in the active cycle (KI67+, **Figure 5B**) and the up-regulation of Nestin+ and Mash1+ progenitor numbers induced by IL-10 (**Figure 5C**), thus indicating that regular cellular differentiation was re-established. In addition, the ERK inhibitor abolished the detrimental effect of IL-10 on the presence of DCX+ neuroblasts, thereby showing that neuronal determination was re-establish when ERK phosphorylation was inhibited. With this experimental approach, no alterations in Olig2 cell numbers was detected in the presence of U0126 (data not shown).

All together, these results show that ERK1/2 activation mediates IL-10 signaling in progenitors of the adult SVZ niche.

### THE DOWN-REGULATION OF STAT3 EXPRESSION REVERSED THE EFFECTS OF IL-10 ON SVZ PROGENITORS

Several inhibitors of STAT3 were evaluated in SVZ-dissociated cultures. However, all of them compromised the viability of the cultures even when used at very low doses, below their pharmacological activity. To overcome this problem and rule out the relevance of STAT3 in IL-10 signaling both *in vivo* and *in vitro*, lentiviral vectors encoding a GFP reporter together with a miRNA-embedded shRNA sequence targeting STAT3 (Lent-GFP-miSTAT3) or a control shRNA (Lent-GFP-miCont) were designed and prepared (see Materials and Methods). The efficiency to silence STAT3 was checked in several primary cultures, including SVZ primary cultures, in which a considerable reduction in total STAT3 levels was observed (see Supplementary Figure S3A) taking into account that the transduction efficiency varied between 30 and 40% (See Supplementary Figure S3B).

**FIGURE 6:**
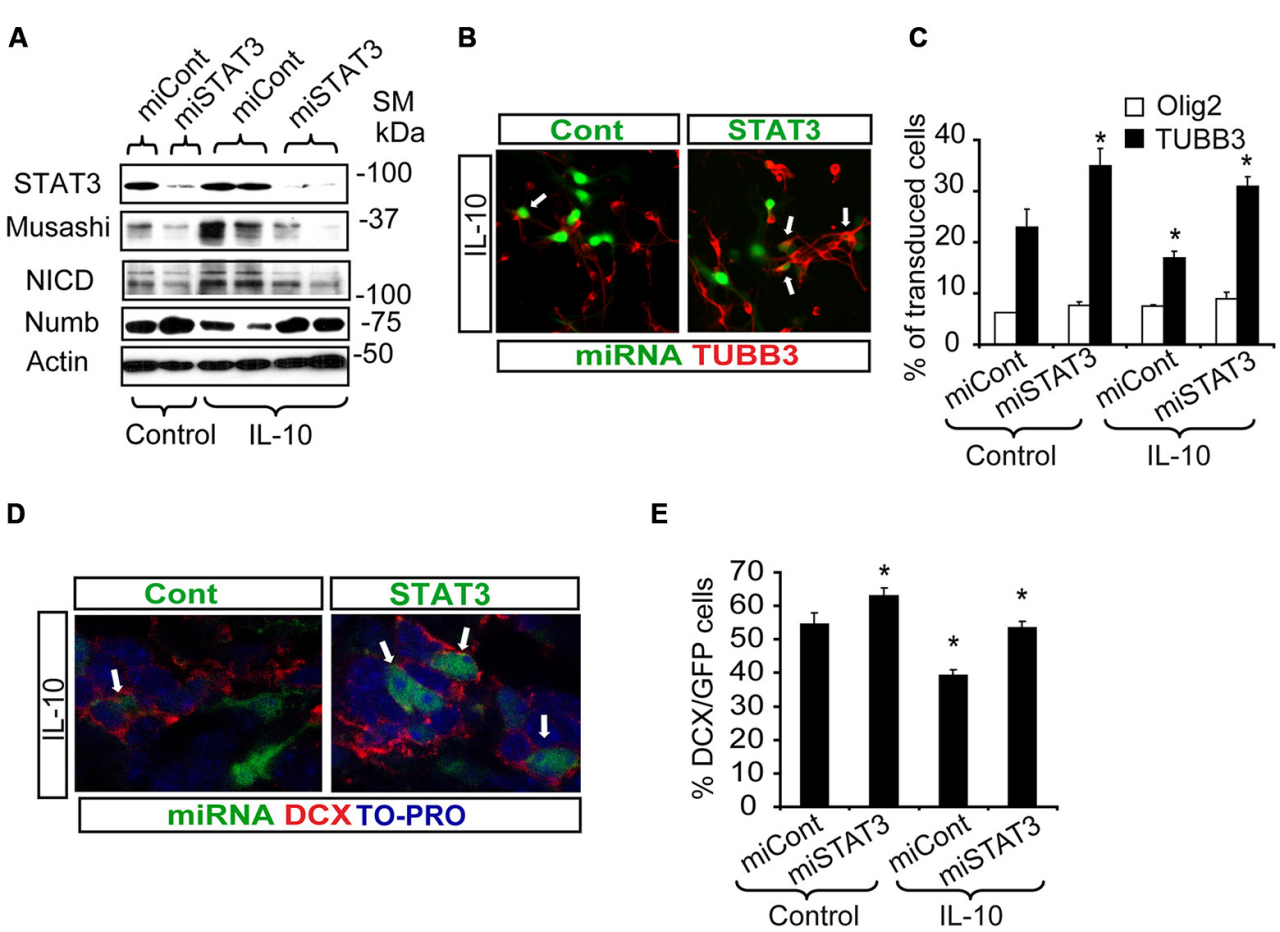
**STAT3 downregulation reverts the effects of IL-10 treatment and improves neurogenesis *in vitro* and *in vivo*. (A)** Western blotting of neural protein expression after lentiviral transduction with Lent-GFP-micont (miCont) or Lent-GFP-miSTAT3 (miSTAT3) treatment during 72 h. miRNASTAT3 prevents the regulation of Musashi, NICD and Numb induced by IL-10 (*n* = 4). **(B)** Pictures of dissociated cultures infected either by miCont or miSTAT3 in the presence of IL-10 stained against TUBB3. STAT3 down-regulation induced an increase in the number of neuroblasts (TUBB3+, red, arrows) among transduced cells (green). **(C)** Graph summarizes the effect of STAT3 downregulation on the numbers of TUBB3+, GFAP+, and olig2+ cells in control situation and after IL-10 incubation. Significant differences in the numbers of TUBB3 positive cells were observed in IL-10-treated cultures by the presence of miSTAT3. Values are expressed as the percentage of control (*n* = 5). **(D)** *In vivo* inhibition of STAT3 expression on SVZ progenitors improves the number of neuroblast-committed cells (DCX+ cells, red) on dorsal SVZ, 7 days after lentiviral particles administration *in vivo*. **(E)** Graph represents the effect of miSTAT3 *in vivo*. The alteration of STAT3 expression increases the presence of DCX+ cells on the dorsal SVZ. Scale bar: 30 μm. Data are represented as mean ± SEM. *P < 0.05.

In SVZ neural cultures, transduction with Lent-GFP-miSTAT3 attenuated the general IL-10-induced expression of genes like Musashi and NICD, as markers of progenitor undifferentiation, and NUMB expression was recovered (**Figure 6A**). Evaluation of some of these markers at the cellular level demonstrated that treatment with IL-10 reduced the number of neuroblasts (TUBB3+ cells, Lent-GFP-miCont cultures, (**Figures 6B,C**), as expected. In contrast, the presence of Lent-GFP-miSTAT3 reverted the effects of IL-10, and the number of neuroblasts (red, TUBB3+ cells; **Figures 6B,C**) among transduced cells was similar to those detected in Lent-GFP-miCont cultures (green, arrows **Figures 6B,C**). In untreated cultures, the down-regulation of STAT3 expression increased neuroblast numbers, thereby suggesting that this molecule was specifically required for neuronal differentiation. In all the experimental conditions, the numbers of Olig2+ cells (**Figure 6C**) and astrocytes (GFAP+ cells, data not shown) were not altered by STAT3 down-regulation. This observation thus indicates that the differentiation of these cells is not dependent on STAT3.

All together, these results support the requirement of STAT3 to mediate the function of IL-10 as a neurogenic factor and its involvement in the regular neuronal differentiation of SVZ progenitors.

Next, we assayed the effect of STAT3 down-regulation in a physiological context. For this purpose, either control or STAT3-shRNA lentiviral vectors were administered *in vivo* in the ctr hemisphere by adding the lentiviruses to the osmotic mini-pump implanted for IL-10 administration. Inhibition of STAT3 expression (Lent-GFP-miSTAT3) improved the determination of the neuronal phenotype previously impaired by IL-10 treatment (**Figures 6D,E**). This finding supports the notion that STAT3 is required for IL-10 activity. All together, our results demonstrate that STAT3 regulates adult neurogenesis *in vivo* and mediates IL-10 function.

## DISCUSSION

The SVZ niche has a particular regional brain localization, allowing a complex network of interactions between neighboring cells, the vasculature, and the CSF. Molecular signals provided by this rich environment regulate cell identity and renewal, and the proliferation and differentiation of the resident progenitors (Merkle et al., 2004). In a previous study, we identified the cytokine IL-10 as a key factor that regulates adult neurogenesis through a mechanism of action independent of its well-known anti-inflammatory properties (Perez-Asensio et al., 2013). In the present study we deciphered the intracellular molecular pathways activated by IL-10 on dorsal SVZ progenitors to exert its anti-neurogenic properties. Our results demonstrate that ERK and STAT3 are activated by IL-10 in Nestin+ progenitors and are required for the anti-neurogenic action exerted by IL-10 on these cells.

The IL receptor signals through the activation of the JAK/STAT pathway in several systems (Matsuda et al., 1999). IL-10-induced STAT3 activation by the JAK1 adaptor is a key element mediating its well-known anti-inflammatory properties (Lois and Alvarez-Buylla, 1994; Matsuda et al., 1999; Levy and Darnell, 2002). During mammalian cerebral cortical development, NSCs present in germinal regions give rise to successive waves of neurons, followed by oligodendrocytes and astrocytes, in a process orchestrated by bone morphogenetic proteins (BMPs) and some cytokines (Bonni et al., 1997; Mehler et al., 2000). Cytokine activation of JAK-STAT signaling controls cell fate during mammalian development and selectively enhances the differentiation of precursors along a glial lineage (Mayer et al., 1994; Bonni et al., 1997; Nakashima et al., 1999).

During CNS development, STAT3 phosphorylation is a dominant signal for glial differentiation in the presence of ligands like CNTF, LIF, Notch, and BMPs (Bonni et al., 1997; Aberg et al., 2001). In addition, STAT3 has been reported to be an indispensable element for the maintenance and survival of embryonic and NSCs (Matsuda et al., 1999; Raz et al., 1999; Foshay and Gallicano, 2008; Ying et al., 2008). STAT3 phosphorylation on Tyr705 by JAKs modulates the transcriptional activation of STAT3. However, it has been described that Notch signaling activates STAT3 independently of the JAK pathway and mediates stem cell expansion (Matsuda et al., 1999; Androutsellis-Theotokis et al., 2006). In the present study, we found that IL-10 induces Ser727 phosphorylation on STAT3 in Nestin+ progenitors, while kinases of the JAK family are not activated by this cytokine. Phosphorylation on Ser727 can be modulated by ERKs and other kinases, such as mTOR. Our results suggest that IL-10-induced STAT3 activation in Nestin+ progenitors is mediated by ERK1/2. The specific activation of this intracellular signaling pathway by IL-10 in the adult SVZ induces notable changes the expression of the Musashi/NUMB/Notch pathway, which regulates neural cell status maintenance versus neuronal differentiation. Notch is a critical element in the self-renewal and maintenance of NSCs (Androutsellis-Theotokis et al., 2006; Andreu-Agullo et al., 2009; Aguirre et al., 2010; Basak et al., 2012). NUMB inhibits Notch signaling by binding to its intracellular domain, directing it into degradation pathways, and it has been implicated in the regulation of some aspects of neuronal differentiation. Musashi leads to the down-regulation of NUMB by binding to its 3^′^ UTR mRNA to inhibit translation (Imai et al., 2001; Li et al., 2003; Petersen et al., 2004; Kuo et al., 2006). IL-10 signaling through ERK1/2 and STAT3 causes marked changes in the NUMB-NOTCH-Musashi pathway, leading to an undifferentiated stage in SVZ Nestin+ progenitors as opposed to differentiation (Perez-Asensio et al., 2013; present data).

Ras/ERK1/2 activity is critical during neurodevelopment and brain tumor formation (Li et al., 2014). Constitutive activation of the MAPK signaling pathway promotes a pro-neural genetic switch and induces glial differentiation in the developing embryonic brain (Samuels et al., 2008; Wang et al., 2012; Li et al., 2014). The NF1 gene is a negative regulator of Ras/ERK1/2 signaling, and NF1 inactivation impairs the balance of glial versus neuronal output in the embryonic and postnatal brain. The neonatal inhibition of ERK1/2 activity reverses the effects caused by NF1 inactivation (Wang et al., 2012). Our data showed that IL-10 activates ERK and promotes a pro-neural stage of Nestin+ progenitors of the adult SVZ, without affecting glial differentiation. However, when ERK1/2 activity was severely reduced by using high concentration of the pharmacological inhibitor *in vitro*, oligodendroglial differentiation was also impaired, independently of IL-10 activity. In contrast, the *in vivo* data showed no changes in the numbers of Olig2 cells when the MAPK inhibitor was used at low concentrations sufficient to prevent the pro-neural state induced by IL-10.

**FIGURE 7:**
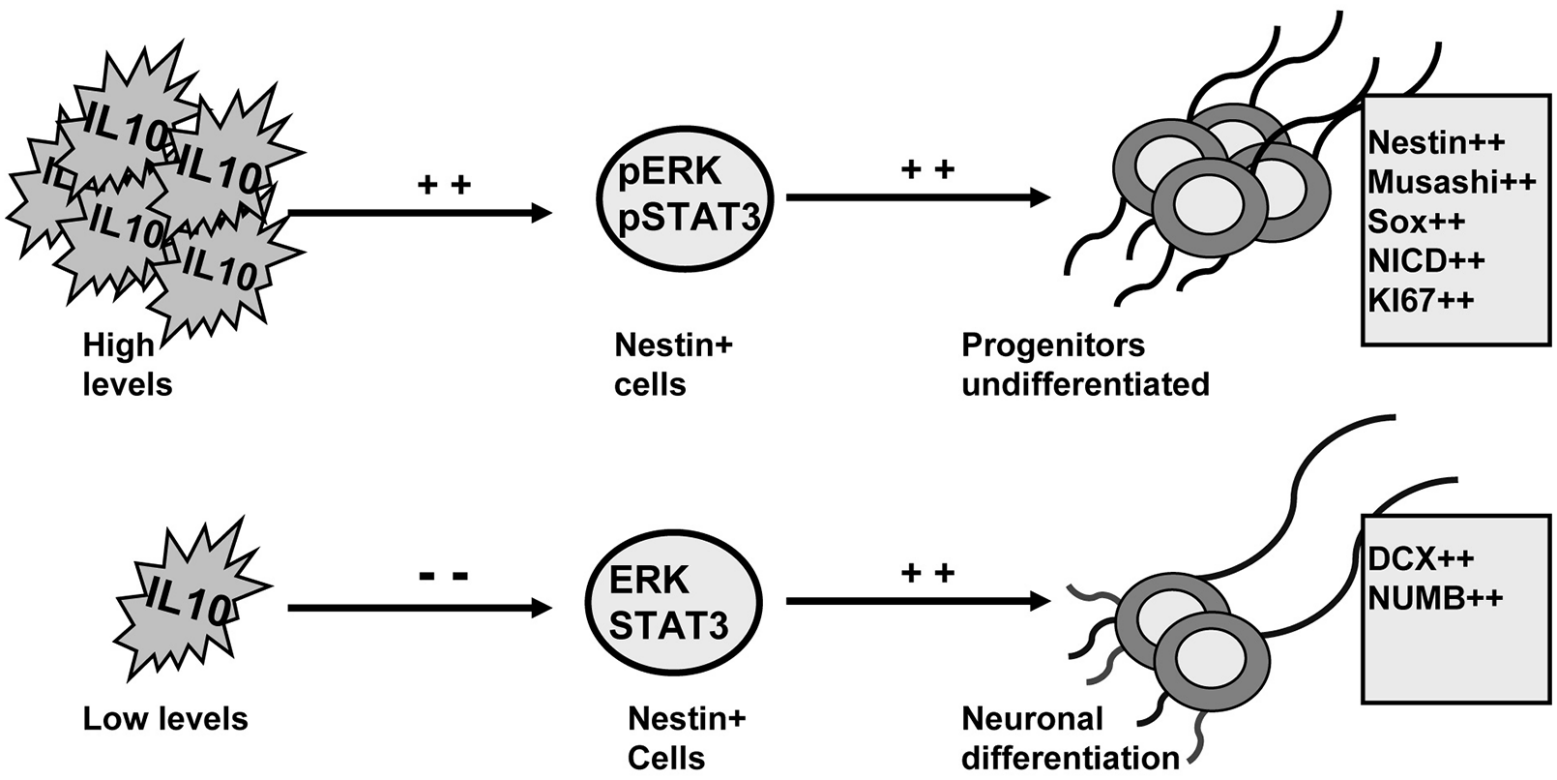
**ERK1/2 and STAT3 mediate the actions of IL-10 to regulate adult neurogenesis on adult SVZ.** Schematic drawing summarizing the findings reported in this work. IL-10 induces ERK1/2 and STAT3ser727 phosphorylation on Nestin+ cells. The activation of both intracellular mediators leads to the accumulation of undifferentiated neural progenitors. Conversely, when IL-10 levels are reduced the activity of ERK and STAT3 is attenuated and neuronal differentiation is induced. The outcome is a higher production of neurons.

IL-10 levels control adult neurogenesis by regulating progenitor differentiation (Perez-Asensio et al., 2013). Here we demonstrate that ERK1/2 and STAT3 are activated by IL-10 and are mediators of the effects of this cytokine in the dorsal SVZ. The induction of ERK1/2 and STAT3 activity by IL-10 promotes an undifferentiated stage in progenitor cells. Conversely, neuronal differentiation is permitted in the presence of IL-10 when the activity of ERK1/2 and STAT3 is compromised (summarized in **Figure 7**).

## AUTHOR CONTRIBUTIONS

LP and MF-N performed experiments and analyzed some data. MF-N, CVdH, and VB develop and produced lentiviral vectors. AMP contributed to the starting of the experiments and has been involved in drafting and revising the manuscript. EP designed and performed experiments, supervised the study, analyzed the data and wrote the manuscript. All authors agree that all the questions related to the accuracy or integrity of the work have been appropriately investigated and resolved, giving final approval of the version to be published.

## Conflict of Interest Statement

The authors declare that the research was conducted in the absence of any commercial or financial relationships that could be construed as a potential conflict of interest.

## Abbreviations

ICV: intracerebroventricular
LV: lateral ventricle
NICD: Notch intracellular domain
NSC: neural stem cell
RMS: rostral migratory stream
SVZ: subventricular zone
TAC: transit amplifying cell
