# Supplementary material for: IL-10 regulates adult neurogenesis by modulating ERK and STAT3 activity
Source: Front Cell Neurosci. 2015 Feb 25;9:57. doi: 10.3389/fncel.2015.00057 (PMC4340210; doi:10.3389/fncel.2015.00057)

Supplementary information

IL-10 regulates adult neurogenesis by modulating ERK and STAT3 activity

Leticia Pereira[1,2], Miriam Font-Nieves[1,2], Chris Van den Haute[3], Veerle Baekelandt[3] , Anna M. Planas[1,2] and Esther[1,2] Pozas*

3 supplementary figures and legends

Supplementary Figure 1

IL-10 administration into femoral vein induces a fast activation of ERK p42-44 on SVZ adult niche.

When IL-10 was administered into femoral vein phosphorylation of ERK1/2 was induced shortly after cytokine administration (30 min) and in a dose dependent manner in living mice (n=5). Actin was use as a loading control.

Supplementary Figure 2

ERK inhibition reduces oligodendroglial differentiation in independency of IL-10 presence

Graph summarizes the effect of U0126 on the numbers of Olig2+ and GFAP+ cells in control situation and after IL-10 incubation. In control and IL-10-treated cultures U0126 reduced the presence of Olig2+ cells, while astrocytes numbers (GFAP+) were unaltered. Values are expressed as the percentage of control (n=5). Data are represented as mean ± s.e.m. * $P < 0.05$.

Supplementary Figure 3

Transduction with Lent-GFP-miSTAT3 on SVZ primary cells robustly reduced STAT3 expression

(A) Western blot shows that transduction of Lent-GFP-miSTAT3 reduces STAT3 expression on primary SVZ cells. Expression of STAT1 was unchanged, actin was used as a loading control (n=3).

(B) Percentage of transduced cells on SVZ dissociated cultures by Lent-GFP-micont or Lent-GFP-miSTAT3 in the presence or absence of IL-10.

Supplementary Fig. S1

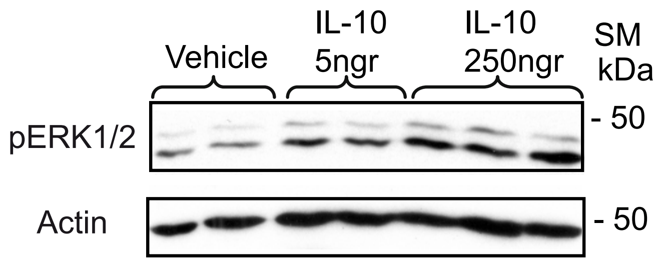

Supplementary Fig. S2

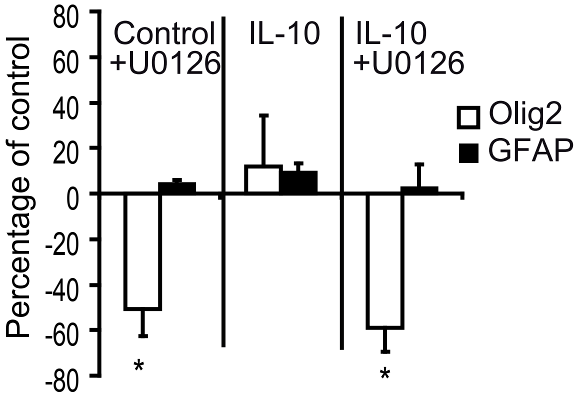

Supplementary Fig. 3

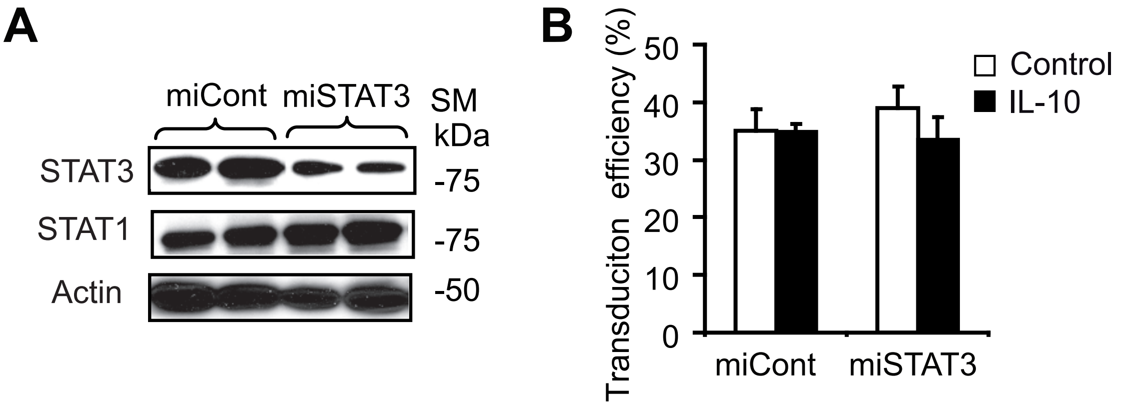

Supplement: Supplementary file 1 [file presentation_1.pdf]
